# Molecular Evidence for the Fitness of Cotton Aphid, *Aphis gossypii* in Response to Elevated CO_2_ From the Perspective of Feeding Behavior Analysis

**DOI:** 10.3389/fphys.2018.01444

**Published:** 2018-11-12

**Authors:** Shoulin Jiang, Yang Dai, Yongqing Lu, Shuqin Fan, Yanmin Liu, Muhammad Adnan Bodlah, Megha N. Parajulee, Fajun Chen

**Affiliations:** ^1^Department of Entomology, College of Plant Protection, Nanjing Agricultural University, Nanjing, China; ^2^Personnel Department, Qingdao Agricultural University, Qingdao, China; ^3^Qidong Agricultural Commission, Qidong, China; ^4^Texas A&M University AgriLife Research and Extension Center, Lubbock, TX, United States

**Keywords:** elevated CO_2_, *Aphis gossypii*, fitness, feeding behavior, molecular evidence

## Abstract

Rising atmospheric carbon dioxide (CO_2_) concentration is likely to influence insect–plant interactions. Aphid, as a typical phloem-feeding herbivorous insect, has shown consistently more positive responses in fitness to elevated CO_2_ concentrations than those seen in leaf-chewing insects. But, little is known about the mechanism of this performance. In this study, the foliar soluble constituents of cotton and the life history of the cotton aphid *Aphis gossypii* and its mean relative growth rate (MRGR) and feeding behavior were measured, as well as the relative transcript levels of target genes related appetite, salivary proteins, molting hormone (MH), and juvenile hormone, to investigate the fitness of *A. gossypii* in response to elevated CO_2_ (800 ppm vs. 400 ppm). The results indicated that elevated CO_2_ significantly stimulated the increase in concentrations of soluble proteins in the leaf and sucrose in seedlings. Significant increases in adult longevity, lifespan, fecundity, and MRGR of *A. gossypii* were found under elevated CO_2_ in contrast to ambient CO_2_. Furthermore, the feeding behavior of *A. gossypii* was significantly affected by elevated CO_2_, including significant shortening of the time of stylet penetration to phloem position and significant decrease in the mean frequency of xylem phase. It is presumed that the fitness of *A. gossypii* can be enhanced, resulting from the increases in nutrient sources and potential increase in the duration of phloem ingestion under elevated CO_2_ in contrast to ambient CO_2_. In addition, the qPCR results also demonstrated that the genes related to appetite and salivary proteins were significantly upregulated, whereas, the genes related to MH were significantly downregulated under elevated CO_2_ in contrast to ambient CO_2_, this is in accordance with the performance of *A. gossypii* in response to elevated CO_2_. In conclusion, rise in atmospheric CO_2_ concentration can enhance the fitness of *A. gossypii* by increasing their ingestion of higher quantity and higher quality of host plant tissues and by simultaneously upregulating the transcript expression of the genes related to appetite and salivary proteins, and then this may increase the control risk of *A. gossypii* under conditions of climate change in the future.

## Introduction

Global atmospheric carbon dioxide (i.e., CO_2_) concentration has continuously risen from about 280 ppm to 408 ppm as on May 2018 (Mauna Loa Observatory: NOAA-ESRL) and future estimations predict an increase up to 550 ppm within a few decades (Pachauri et al., 2014). Rising CO_2_ has been an aspect of global climate change, being one great concern for the scientific community, owing not only to its “greenhouse effects” (Tubiello et al., 2000) but also its influences on the physiological and biochemical characters of the plant (Ainsworth and Rogers, 2007). The gaseous form of CO_2_ is the direct substrate for photosynthesis in plants (Ziska, 2008), which shows typical increases of photosynthetic rate, biomass, leaf area, and carbon (C): nitrogen (N) ratio, especially in C_3_ crops (During photosynthesis, “C” in CO_2_ is fixed directly to “C_3_” in plants, such as, rice and cotton.) (Ainsworth and Long, 2005; Chen et al., 2005a; Ainsworth et al., 2007). Generally, elevated CO_2_ alters plant chemistry by the assimilation and reassignment of C and N resources within plant tissues (Couture et al., 2010). Based on evidence provided by Chen et al. (2004) and Oehme et al. (2013), in spring wheat (*Triticum aestivum*), elevated CO_2_ significantly increases the soluble components of plant tissues, such as free amino acids (FAAs), soluble proteins, and glucose. Similar results were also demonstrated by cotton (*Gossypium hirsutum*) plants, that is, elevated CO_2_ significantly enhanced foliar soluble matters, including soluble sugars, FAAs, and fatty acids, which had further positive effects on the population growth of the cotton aphid, *Aphis gossypii*, in response to elevated CO_2_ (Jiang et al., 2016). As noted in various studies, elevated CO_2_ directly affects the primary and secondary metabolites of host plants, which, in turn, indirectly alter the performance of herbivorous insects (Couture et al., 2010; Guo et al., 2014a; Jiang et al., 2016).

Sap-sucking insects have shown consistently more positive responses in fitness to elevated CO_2_ concentrations (Sun et al., 2015) than those shown by leaf-chewing insects (Bezemer and Jones, 1998). They feed exclusively on the phloem of their host plants (Douglas, 2003), and the phloem sap mainly contains sucrose (up to 80–85% of the organic components) and soluble proteins (SPs) (Avigad and Dey, 1997). Moreover, sucrose is also recognized as an important transportable sugar in most plant species and as the most effective phagostimulant for herbivorous insects (Hawker, 1985). Moreover, the concentration of SPs in the phloem sap is regarded as a key factor for identifying the nutritional quality of host plants by aphids (Nowak and Komor, 2010). Therefore, since elevated CO_2_ inevitably alters plant metabolites, the performance of sap-sucking insects is affected by the bottom-up effects of the host plants in terms of nutritional status (Awmack and Leather, 2002). For example, rising atmospheric CO_2_ increases the population growth of *Acyrthosiphon pisum*, owing to enhanced food ingestion and good food-quality plasticity; specifically, it increases amino acids’ concentration and other nutrient components in leaves and phloem sap (Guo et al., 2013). Furthermore, the impact of elevated CO_2_ on the growth, development, and fecundity of the cotton aphid *A. gossypii* was mainly indirect, which is affected by the nutritional status of the plant (Chen et al., 2005b; Jiang et al., 2016).

Electrical penetration graph (EPG) technique, which monitors the stylet penetration behavior via variation in electrical recording signals, is a well established and effective experimental method to quantify the sap-feeding behavior of aphids (McLean and Kinsey, 1964; Tjallingii, 1988, 1990; Jiang et al., 2015). Our previous study indicated that elevated CO_2_ promoted the ingestion efficiency of the cotton aphid *A. gossypii* and simultaneously increased the leaf turgor and foliar soluble constituents of cotton plants (Jiang et al., 2015). Although the feeding behavior of aphids in response to elevated CO_2_ has been well established, the underlying molecular mechanism of elevated CO_2_-induced changes in the ingestion in aphids remains largely unknown. It has been documented that the feeding behavior of insects is regulated by neuropeptide F (i.e., NPF) and angiotensin-converting enzymes (i.e., ACE) related to appetite (Nassel and Wegener, 2011; Wang et al., 2015). Wu et al. (2003) reported that the expression of NPF was high in the larvae of *Drosophila melanogaster* that were attracted to food, whereas its downregulation coincided with food aversion and hyperactivity of older larvae; the the over-expression of NPF in older larvae conversely promoted feeding and suppressed hypermobility and excessive behaviors. Numerous invertebrates, for example, *Litopenaeus vannamei* and *Melicertus marginatus* (Christie et al., 2011), *Caenorhabditis elegans* (de Bono and Bargmann, 1998), *Periplaneta americana* (Mikani et al., 2012), *Latrodectus hesperus* (Christie, 2015), and *Schistocerca gregaria* (Van Wielendaele et al., 2013), exhibit the fact that NPF has a function in the modulation of feeding behavior. Likewise, it was demonstrated that ACE modulates the aphid–plant interactions by affecting feeding behavior and survival of aphids, through evidence obtained from the knockdown of ACE genes (Wang et al., 2015). Previous studies showed that the salivary sheath protein and C002 play a critical role in the process of stylet penetration and food ingestion in aphids (Mutti et al., 2008; Bos et al., 2010; Abdellatef et al., 2015). Here, the question is what are the underlying molecular mechanisms that elicit the positive responses in the fitness of sap-sucking insects to elevated CO_2_.

The Cotton aphid, *A. gossypii*, as a typical phloem-feeding insect, is known as one of the most problematic insect pests of cotton plants worldwide. In this study, an EPG experiment was carried out with cotton (*G. hirsutum*) plants and the cotton aphid *A. gossypii* under ambient and elevated CO_2_ in open-top chambers; simultaneously, an assay to identify the foliar soluble constituents of cotton plants and the molecular biology analysis of the genes related to appetite and salivary proteins of cotton aphids were conducted. The purpose was to examine the effects of elevated CO_2_ on stylet ingestion and fitness of phloem-feeding insects on host plants as well as elucidate the molecular mechanisms of feeding behavioral response of phloem-feeders when the host plant is exposed to rising atmospheric CO_2_ concentrations.

## Materials and Methods

### CO_2_ Levels and Condition Setting

This study was conducted in six identical electronically controlled growth incubators (GDN-400D-4/CO_2_; Ningbo Southeast Instrument Co., Ltd., Ningbo, China) with a gas-tank system that maintained the desired CO_2_ concentration. In these growth incubators, a periodic regime was maintained at 26°C and 70% RH during the day, 25°C and 70% RH at night, and L14: D10 photoperiod with light at 20000 Lux supplied by LED lamps. The CO_2_ concentrations in the three growth incubators mentioned above were set at the current atmospheric CO_2_ level (i.e., 400 ppm), and the rest of the three growth incubators were set at an elevated CO_2_ level (i.e., 800 ppm), which was the predicted CO_2_ level at the end of the 21st century (Mastrandrea et al., 2011). During the experiment, the six growth incubators were alternated by switching CO_2_ concentration rates as well as swapping the entire content of each growth incubator every 5 days in order to equalize the possible bias on the cotton plants and aphids due to the incubator-specific growth conditions.

### Host Plants and Cotton Aphids

Cotton (cv. C111) was planted in white plastic pots (12 cm diameter, 15 cm high) filled with nutritional soil (Xingnong Organic Fertilizer Co., Ltd., Zhenjiang, China). After the seedlings’ emergence, cotton plants were thinned to one plant per pot and exposed to the above mentioned (about 400 ppm) and elevated (about 800 ppm) CO_2_ conditions. The cotton plants were watered moderately every day; no additional chemical fertilizers or insecticides were used. At least 60 pots were randomly placed in each growth incubator (i.e., a total of 180 pots of cotton plants per CO_2_ treatment) and re-randomized once a week to minimize position effects within each growth incubator.

The colony of the apterous cotton aphid *A. gossypii* used in this study was provided by Prof. Xiangdong Liu from the Department of Entomology, Nanjing Agricultural University. To obtain a standardized aphid colony for this experiment, only one clone in this colony was selected to establish an experimental population of *A. gossypii*. The colony was maintained on 35- to 60-day-old cotton seedlings planted in the same white plastic pots filled with the same nutritional soil in the same electronically controlled growth incubators mentioned above for the following experiments.

### Foliar Soluble Constituents of Cotton Seedlings

For the quantitative analysis of foliar soluble nutrition of cotton seedlings, 30 fully expanded leaves on the third to fourth main stem nodes were randomly selected and excised from the potted cotton seedlings in the above mentioned growth incubators of ambient and elevated CO_2_ treatments, respectively. Cotton leaves were ground into a fine powder with a mortar and pestle in liquid nitrogen. For the determination of foliar FAAs, the leaf powder (accurately weighed 200–300 mg) was transferred to a 50 ml centrifuge tube, and then, it was diluted to a 10 ml solution by 0.02 mol/L HCl solution. The extraction buffer was sonicated for 15 min at 4°C, and then centrifuged for 15 min at 4,000 rpm/min (RCF = 1503 g) at 4°C to obtain the supernatant containing FAAs, 700 ml of the supernatant was transferred to a 1.5 ml microtube for deproteinization by an equal volume of 4% sulfosalicylic acid solution, then centrifuged for 15 min at 4,000 rpm/min (RCF = 1503 g) at 4°C. The supernatants of the all the samples were individually filtered through 0.22 μm hydrophilic membranes, and, finally, the measurement of FAA concentrations was performed using an automatic FAA analyzer (L-8900; Hitachi High-Technologies Corporation, Tokyo, Japan). The values of FAAs were expressed as mg/g fresh weight.

The above obtained leaf powder was collected, approximately 30–40 mg fresh weight was transferred into a 1.5 ml microtube, and 0.9% saline was used as an extraction buffer at a ratio of 1:9 (tissue weight in g and buffer volume in ml) for the measurement of the foliar SP content. The supernatant of extraction buffer was used as a protein solution for the following test. The foliar SP content was determined by following the instructions of the corresponding diagnostic kit A045-2 (Jiancheng Bioengineering Institute, Nanjing, China). For sucrose determination, 30–40 mg of above obtained leaf power was collected in a 5 ml centrifuge tube with distilled water at a ratio of 1:10 (tissue weight in g and buffer volume in ml), and the mixture was boiled for 10 min and centrifuged at 4,000 rpm/min (RCF = 1503 g) for 10 min. The supernatants were used for assaying the foliar sucrose content according to the corresponding diagnostic kit for the determination of plant sucrose content (Jiancheng Bioengineering Institute, Nanjing, China). There were three replicates for assaying the foliar contents of soluble constituents (including FAA, SP, and sucrose) of cotton seedlings.

### Aphid Infestation

#### Life History Parameters of Cotton Aphids

A total of 45 newborn first instar nymphs were selected from the above mentioned aphid colony of *A. gossypii* and individually reared on fully expanded leaves, which were excised from the 35- to 60-day-old cotton seedlings grown under ambient and elevated CO_2_, respectively, in glass culture dishes (150 mm in diameter; one nymph per leaf × one leaf per dish × 15 dishes per growth incubator ×3 growth incubator per CO_2_ treatment). Aphid nymphs were monitored twice a day to record molting until they developed into adults. The exuvia was removed, and the ecdysis time was recorded to quantify the nymphal duration of *A. gossypii*. Moreover, the number of offsprings laid per adult was recorded twice a day, and all the nymphs were removed until the adult aphid died to determine fecundity. The life history parameters of the reproductive period and adult longevity were also, finally, calculated and recorded. In this experiment, eight nymphs of the ambient CO_2_ treatment and four nymphs of the elevated CO_2_ treatment died in the rearing process, and actually, there were 37 and 41 individuals of *A. gossypii* in the treatments of ambient and elevated CO_2_, respectively. But, for assessing survival rate, we added data to 45 replicates in two CO_2_ treatments.

#### Mean Relative Growth Rate (MRGR)

A total of 30 newborn first instar aphids were randomly selected from the above mentioned aphid colony and weighed (i.e., W1) using a precision scale with an accuracy of ±1 μg (Mettler Toledo XP6, Switzerland) and then individually reared using the same protocol for the measurement of life history parameters of *A. gossypii* (i.e., one nymph per leaf × one leaf per dish × 10 dishes per growth incubator × 3 growth incubator per CO_2_ treatment). These tested aphid nymphs were reweighed (i.e., W2) by using the same precision scale after 5 days of rearing, and the mean relative growth rate (i.e., MRGR) of *A. gossypii* nymphs was calculated based on the method described by Hodge et al. (2005): MRGR = (lnW2 - lnW1)/t, where W1 is the initial weight, W2 is the final weight, and t is the rearing time (here, 5 days) of *A. gossypii* nymphs. In this experiment, two nymphs of the ambient CO_2_ treatment and five nymphs of the elevated CO_2_ treatment died in the rearing process, and actually, there were 23 and 20 individuals of *A. gossypii* in the treatments of ambient and elevated CO_2_, respectively.

#### Electrical Penetration Graphs (EPG) to Monitor Aphid Feeding

To monitor the feeding behavior of the cotton aphid *A. gossypii*, 300 newborn first instar nymphs were randomly selected from the above mentioned aphid colony and reared on fully expanded leaves, which were excised from cotton seedlings grown under ambient and elevated CO_2_ conditions, respectively, in 30 culture dishes (150 mm in diameter; 10 nymphs per leaf × one leaf per dish × 10 dishes per growth incubator × 3 growth incubator per CO_2_ treatment). Once the newborn adult aphids emerged, they were randomly selected and used for the following EPG test.

The feeding activities of the cotton aphid *A. gossypii* were studied by using a Giga-8 DC-EPG amplifier system with 1 GΩ input impedance, 50× amplification, and <1 pA input bias current (Wageningen University, Wageningen, Netherlands). The above mentioned newborn adult aphids were individually connected to a gold wire (0.5 mm diameter, 3 cm long) with conductive silver glue on their dorsum. After 1 h of starvation, the wired adult aphids were carefully placed on the abaxial surface of the fully expanded leaf in the same culture dishes mentioned above, and the other side of the gold wire was connected to the amplifier of the Giga-8 DC-EPG amplifier system. The experiment was conducted in a greenhouse at 26.5 ± 1°C, 70 ± 10% RH, and L14:D10 photoperiod. Based on previous studies, probing behavior was continuously recorded for 5 h, and the 4-h effective records (which contained enough effective information for data analysis) from the beginning of the feeding test were analyzed using the EPG Stylet software (EPG Systems, Wageningen, Netherlands). All recorded signals were analyzed, including non-penetration period (i.e., the NP waveform indicating aphid walking and stylet not probing the host substrate), pathway phase (i.e., the C waveform indicating aphid stylet probing the host substrate to locate the feeding site), phloem phase (i.e., the E waveform, including two events: the E1 waveform showing salivation into phloem sieve elements; the E2 waveform showing ingestion of the phloem content), and xylem phase (i.e., the G waveform indicating ingestion of the xylem sap). In this study, there were eight types of EPG recordings, including the waveforms of NP, C, E1, E2, G, first E1, first E2, and E2 > 8 min (seen in Table 1). The waveform parameters of the first E1 waveform and the first E2 waveform indicated the duration of the first occurrence of E1 and E2, respectively; the waveform of E2 > 8 min indicated sustained phloem ingestion for more than 8 min (Kimmins and Tjallingii, 1985; Davis and Radcliffe, 2008).

**Table 1:** The electrical penetration graphs (EPG) of the cotton aphid *Aphis gossypii* and the respective correlated stylet penetration activities.

EPG waveform	Definition
NP	Non-penetration period
C	Stylet pathway activity (salivary sheath deposition)
E1	Saliva secretion to phloem tissues
E2	Ingestion from phloem tissues
G	Xylem ingestion
First E1	The first occurrence of E1
First E2	The first occurrence of E2
E2 ≥ 8 min	Sustained phloem ingestion for more than 8 min

### RNA Preparation and Reverse Transcription

The newborn adults of *A. gossypii* sampled for the molecular test were randomly selected from the tested adult aphids used for the above mentioned EPG test. Once the newborn adults emerged, 20 of them were randomly collected from each growth incubator and mixed as one biological replicate, and there were three biological replicates for the treatments of ambient and elevated CO_2_, respectively. Total RNA was extracted from sampled newborn adult aphids by using the TRIzol^®^ reagent (Invitrogen). The concentration and quality of samples were determined by using the NanoDrop^TM^ spectrophotometer (Thermo Scientific) and 1.5% agarose gel electrophoresis. The first-strand complementary cDNA templates were synthesized with 100 ng of total RNA by using the PrimeScript^TM^ RT reagent Kit with gDNA Eraser (TaKaRa, Japan). Reverse transcriptase reactions were performed in a 20 μl final volume reaction.

### Real-Time PCR Analysis

Each cDNA product was diluted from 5× to 80× by diluting twice using RNase-free dH_2_O, in order to make the Ct value to fall within the suitable range of 15–35 based on preliminary experiments. For fluorescence-based quantitative real-time PCR (qRT-PCR), 2 μl of cDNA dilution (100 ng/μl) and 0.2 μM of primers were used in 1× SYBR^®^ *Premix Ex Taq* (TaKaRa) with the 7500 Real-Time PCR Detection System (Applied Biosystems), following the supplier’s instructions. Reactions were performed in a 20 μl final volume. Specific primers for testing the genes were designed by Beacon Designer^TM^ 7.9 software, and the housekeeping gene *RPL* was used as the internal standard to analyze the expression levels of target genes, including appetite related genes [i.e., NPF and ACE], salivary protein genes [i.e., C002a, C002b and salivary sheath protein (SHP)], molting hormone (MH) gene (i.e., CYP314A1) and juvenile hormone genes (i.e., JHAMT and JHEH) of the cotton aphid *A. gossypii*, All the primers used for the qRT-PCR test are shown in Table 2. Quantification of the transcript levels of target genes was conducted by following the 2^-ΔΔCt^ normalization method. The expression levels of the internal control gene were examined in every PCR plate to eliminate systematic errors. Four biological replicates were made for each treatment in the qRT-PCR analysis, and each biological replicate contained three technical repeats.

**Table 2:** The primers used for the qRT-PCR analysis of the related target genes of neuropeptide F (NPF), angiotensin converting enzyme (ACE), salivary proteins (C002a and C002b), salivary sheath protein (SHP), molting hormone (MH), and juvenile hormone (JHAMT and JHEH) of the cotton aphid *A. gossypii*.

Primer		Sequence	Description
RPL	Forward	TGCCGGAGTCTGTACTCAA	Housekeeping gene
	Reverse	TCACACCACGAATACGCA	
NPF	Forward	CTATCACAACACCGAGATTAC	Neuropeptide F
	Reverse	AACAGCATGTCATACAAGTC	
ACE	Forward	AGTTCAATGCCTCAATCT	Angiotensin converting enzyme
	Reverse	TAATCCTATAATCTTGTCTGTTG	
C002a	Forward	CCAAGATTAGAGCACGACT	Salivary protein
	Reverse	AAATGTCTAAAGAAACGTCCA	
C002b	Forward	CCGATTAGCCAGAGTGTT	Salivary protein
	Reverse	TGGAAGGAGTGTTGGTAAG	
SHP	Forward	CCTTGTGATTCTACCGATT	Salivary sheath protein
	Reverse	AGCGACCGTATATTCTCT	
MH	Forward	GCAGCGTGTTCGTATCTA	Molting hormone
	Reverse	TTATTCCAGCGGCAATGTA	
JHAMT	Forward	CAGTTGGTTGGTGTTGATAA	Juvenile hormone-III synthase
	Reverse	GCATACTACGCAAGGAATC	
JHEH	Forward	TTTCCGAACGAAATACCGAT	Juvenile hormone epoxide hydrolase
	Reverse	ATCTCGTAAACTGTCGACCA	

### Data Analysis

Statistical analysis of all data was performed by using the SPSS v.20.0 software (IBM Corporation, Armonk, NY, United States). One-way analyses of variance (ANOVAs) were used to analyze the effects of CO_2_ levels on the foliar contents, on the insect life history parameters and feeding behavior, and on the relative transcript levels of the target genes. Also, the least significant difference (LSD) test was used to analyze the significant differences between the treatments of ambient and elevated CO_2_ at *P* < 0.05. Survival data were calculated using the Kaplan–Meier survival curve and were compared using the log-rank test with a significance threshold of *P* < 0.05. Each experiment was compared with a control group, and all experiments were conducted independently at least three times.

## Results

### Effects of Elevated CO_2_ on the Foliar Contents of Soluble Constituents of the Cotton Seedlings

**FIGURE 1:**
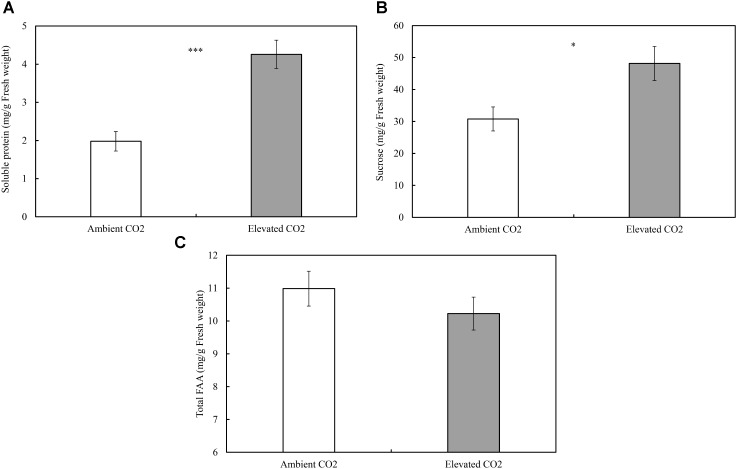
Impacts of elevated CO_2_ on the foliar contents of soluble constituents of cotton seedlings (**A**: soluble proteins; **B:** sucrose; **C**: total free amino acids; asterisks indicate a significant difference between the treatments of ambient and elevated CO_2_ by the LSD test at *P* < 0.05).

The levels of CO_2_ significantly affected the contents of foliar SPs (ie., SPs; *F* = 25.59, *P* ≤ 0.001; Figure 1A) and sucrose (ie., sucrose; *F* = 7.13, *P* ≤ 0.05; Figure 1B), whereas these levels failed to significantly affect the content of total FAAs (ie., FAA; *F* = 1.09, *P* ≥ 0.05; Figure 1C). The order of increase in SPs and sucrose was over 115% and 56% (Figure 1), respectively, in elevated CO_2_ treatment when compared with ambient CO_2_ treatment (*P* < 0.05; Figures 1A,B).

**FIGURE 2:**
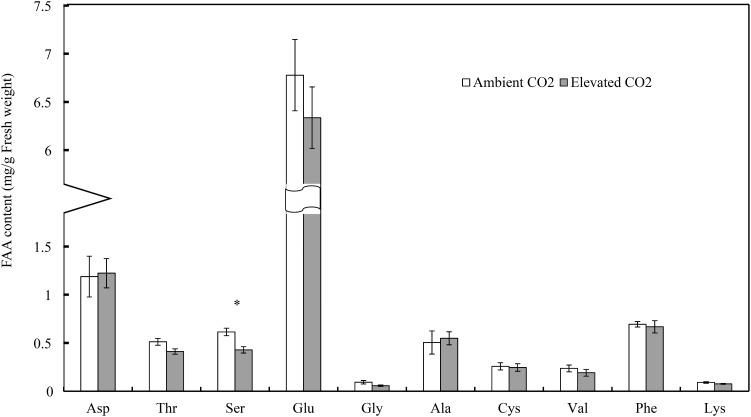
Impacts of elevated CO_2_ on the contents of different types of foliar free amino acids of cotton seedlings (Asterisks indicate a significant difference between the treatments of ambient and elevated CO_2_ by the LSD test at *P* < 0.05).

Moreover, CO_2_ levels significantly affected the foliar serine (Ser) content (*F* = 13.54, *P* < 0.01), whereas these levels failed to significantly affect the contents of other FAAs (*F* ≤ 4.90, *P* ≥ 0.05; Table 3). Compared with ambient CO_2_, elevated CO_2_ significantly decreased the foliar Ser content of cotton seedlings (-30.23%; Figure 2).

**Table 3:** One-way analyses of variances (ANOVAs) for the effects of CO_2_ levels (i.e., ambient vs. elevated) on the foliar contents of soluble constituents of cotton seedlings, and the transcript levels of the target genes related to growth, development, and fecundity of the cotton aphid *A. gossypii* fed on the fully expanded leaves excised from the 35- to 60-day-old cotton seedlings grown under ambient and elevated CO_2_ conditions.

Measured indexes		*F*-Values	*P*-Values
Cotton seedlings (mg/g fresh	Foliar soluble proteins	25.59	0.000***
weight)	Foliar sucrose	7.13	0.018*
	Foliar free amino acids (FAA)	1.09	0.328
Foliar FAA (mg/g fresh weight)	Asp	0.02	0.895
	Thr	4.90	0.058
	Ser	13.54	0.006**
	Glu	0.82	0.392
	Gly	3.36	0.104
	Ala	0.10	0.762
	Cys	0.05	0.83
	Val	0.81	0.395
	Phe	0.14	0.722
	Lys	2.13	0.183
Transcript levels of target genes	Neuropeptide F (NPF)	10.65	0.017*
in *A. gossypii*	Angiotensin converting enzyme (ACE)	11.62	0.014*
	Salivary protein (C002a)	2.87	0.141
	Salivary protein (C002b)	7.89	0.031*
	Salivary sheath protein (SHP)	34.57	0.001**
	Molting hormone (MH: CYP314A1)	9.32	0.022*
	Juvenile hormone (JHAMT)	1.31	0.295
	Juvenile hormone (JHEH)	5.30	0.061

^∗^*P < 0.05, ^∗∗^*P* < 0.01, ^∗∗∗^*P* < 0.001*.

### Effects of Elevated CO_2_ on the Growth, Development, Fecundity, and Survival Rate of the Cotton Aphid *A. gossypii*

The levels of CO_2_ failed to significantly affect the development duration of the first, second, and fourth instar nymphs, total nymphal stages (*F* ≤ 3.58, *P* ≥ 0.062), reproductive period (*F* = 0.07, *P* > 0.05), whereas these levels significantly affected the development duration of the third instar nymph (*F* = 4.07, *P* < 0.05), adult longevity (*F* = 4.95, *P* < 0.05), the whole life span (*F* = 5.02, *P* < 0.05), the fecundity (*F* = 4.23, *P* < 0.05), and the MRGR (*F* = 27.69, *P* < 0.001) of *A. gossypii* (Table 4).

**Table 4:** Mean (±SE) values of the development indexes (including nymph duration, adult longevity, and whole life span), fecundity (including number of offsprings laid per adult and reproductive period), and mean relative growth rate (MRGR) of the cotton aphid *A. gossypii* fed on the fully expanded leaves excised from the 35- to 60-day-old cotton seedlings grown under ambient and elevated CO_2_ conditions.

Measured indexes	*df*	CO_2_ levels		One-way ANOVAs	
		Ambient CO_2_	Elevated CO_2_	*F*	*P*
Nymph duration (days)					
The first instar	1, 76	1.59 ± 0.033	1.72 ± 0.055	3.58	0.062
The second instar	1, 76	1.08 ± 0.041	1.12 ± 0.042	0.48	0.491
The third instar	1, 76	1.11 ± 0.034	1.02 ± 0.024	4.07	0.047*
The fourth instar	1, 76	1.24 ± 0.042	1.20 ± 0.039	0.72	0.399
Total nymph stage	1, 76	5.03 ± 0.061	5.06 ± 0.084	0.10	0.749
Adult longevity (days)	1, 76	17.84 ± 0.922	20.38 ± 0.696	4.95	0.029*
Whole life-span (days)	1, 76	22.86 ± 0.905	25.44 ± 0.725	5.02	0.028*
Reproductive period (days)	1, 76	11.91 ± 0.489	12.09 ± 0.480	0.07	0.794
Number. of offspring laid per adult	1, 76	52.14 ± 2.222	57.56 ± 1.506	4.23	0.043*
Mean relative growth rate (MRGR)	1, 41	0.54 ± 0.007	0.60 ± 0.009	27.69	0.000***

*Asterisks indicate a significant difference between the treatments of ambient and elevated CO_2_ by one-way ANOVAs at *P* < 0.05. Different lowercase letters indicate significant difference between the treatments of ambient and elevated CO_2_ by the LSD test at *P* < 0.05*.

**FIGURE 3:**
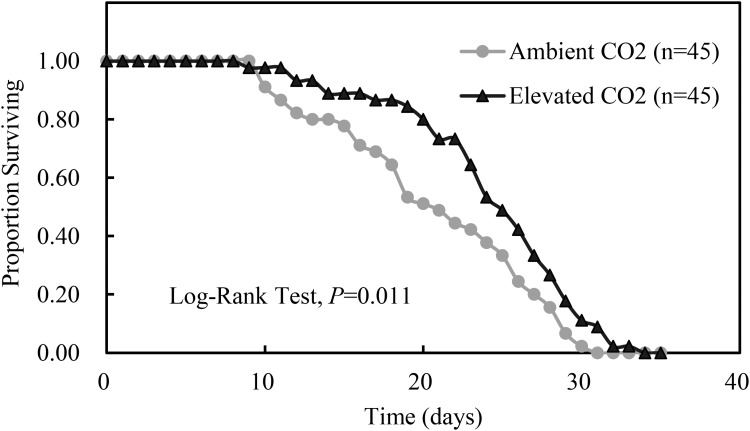
Kaplan–Meier survival curves of *Aphis gossypii* fed on the cotton plant under different CO_2_ levels (ambient vs. elevated) (The significant difference between the treatments of ambient and elevated CO_2_ were obtained by log-rank test at *P* < 0.05).

Compared with ambient CO_2_, elevated CO_2_ significantly shortened the development duration of the third instar nymph by 7.56% (*P* < 0.05) and significantly prolonged the adult longevity and whole life span of *A. gossypii* by 14.24% and 11.26% (*P* < 0.05), respectively, and simultaneously enhanced the number of offsprings per adult and the MRGR of *A. gossypii* by 10.41% and 10.80%, respectively (*P* < 0.05; Table 4). The survival rate of *A. gossypii* from the newborn stage to the death of adult maintained under elevated CO_2_ condition was significantly longer (*P* = 0.011), 24.42 ± 0.87 days, than that seen under ambient CO_2_ condition 20.82 ± 0.98 days (Figure 3).

### Impacts of Elevated CO_2_ on the Feeding Behavior of the Cotton Aphid *A. gossypii*

The EPG data were used to infer possible changes in feeding behavior of *A. gossypii* under ambient and elevated CO_2_ conditions. The data analysis (Table 5) indicated that CO_2_ levels significantly affected the frequency of G phase (*F* = 5.81, *P* < 0.05) and the mean time from the start of the EPG experiment to the first E1 waveform (*F* = 6.77, *P* < 0.05) and the first E2 waveform (*F* = 4.76, *P* < 0.05), whereas these levels failed to significantly affect the frequency of the other EPG waveforms (*F* ≤ 0.81, *P* ≥ 0.38) or the total duration of the EPG waveforms (*F* ≤ 2.89, *P* ≥ 0.10) of the cotton aphid *A. gossypii*.

**Table 5:** Mean (±SE) values of the feeding behavior parameters of the cotton aphid *A. gossypii* fed on fully expanded leaves excised from the 35- to 60-day-old cotton seedlings grown under ambient and elevated CO_2_ conditions.

Measured indexes of the EPG waveforms	*df*	CO_2_ levels		One-way ANOVAs	
		Ambient CO_2_	Elevated CO_2_	*F*	*P*
Mean frequency of the EPG waveforms					
NP	1, 27	8.8 ± 1.5	8.6 ± 1.4	0.01	0.91
C	1, 27	13.2 ± 2.3	12.8 ± 1.9	0.02	0.89
E1	1, 27	6.3 ± 1.2	7.8 ± 1.0	0.81	0.38
E2	1, 27	4.1 ± 0.9	4.1 ± 0.8	0.004	0.95
G	1, 27	3.9 ± 1.2	0.9 ± 0.3	5.81	0.023*
Mean total duration of the EPG waveforms (min)					
NP	1, 27	7.8 ± 3.2	5.2 ± 1.7	0.47	0.49
C	1, 27	106.6 ± 11.4	114.5 ± 13.9	0.20	0.66
E1	1, 27	25.0 ± 6.6	27.1 ± 6.4	0.06	0.82
E2	1, 27	54.9 ± 15.9	73.0 ± 17.1	0.61	0.44
E2 ≥ 8 min	1, 27	47.8 ± 15.0	61.9 ± 16.8	0.40	0.54
G	1, 27	45.9 ± 12.6	20.1 ± 7.9	2.89	0.10
Mean time from the start of the EPG experiment to the E1 and E2 waveforms (min)					
The first E1	1, 27	72.1 ± 10.6	36.8 ± 8.2	6.77	0.015*
The first E2	1, 27	99.2 ± 15.9	59.2 ± 8.3	4.76	0.038*

*NP, non-penetration period; C, stylet pathway activity; E1, saliva secretion to phloem tissues; E2, ingestion from phloem tissues; G, xylem ingestion; The first E, the first occurrence of E1; The first E2, the first occurrence of E2; E2 ≥ 8 min, sustained phloem ingestion for more than 8 min. Asterisks indicate a significant difference between the treatments of ambient and elevated CO_2_ by one-way ANOVAs at *P* < 0.05*.

In contrast to ambient CO_2_, elevated CO_2_ significantly reduced the frequency of the G waveform by 75.99% (*P* < 0.05) and significantly shortened the time from the start of the EPG experiment to the E1 and E2 waveforms by 48.95% and 40.36%, respectively (*P* < 0.05; Table 5). Moreover, an increase in the total duration of the E2 (+33.12%) and E2 ≥ 8min (+29.58%) waveforms was found for the elevated CO_2_ treatment in contrast to the ambient CO_2_ treatment, respectively (*P* > 0.05; Table 5).

### Impacts of Elevated CO_2_ on the Expression of the Target Genes Related to Growth, Development, Reproduction, and Feeding of the Cotton Aphid *A. gossypii*

The levels of CO_2_ significantly affected the expression levels of the appetite related genes of NPF (*F* = 10.65, *P* < 0.05) and ACE (*F* = 11.62, *P* < 0.05), the salivary protein genes of C002b (*F* = 7.89, *P* < 0.05) and SHP (*F* = 34.57, *P* < 0.01), and the MH gene of CYP314A1 (*F* = 9.32, *P* < 0.05), whereas these levels failed to significantly affect the expression levels of the salivary protein gene of C002a (*F* = 2.87, *P* > 0.05), the JH genes of JHAMT (*F* = 1.31, *P* > 0.05) and JHEH (*F* = 5.30, *P* > 0.05) in the cotton aphid *A. gossypii* fed on fully expanded leaves excised from the 35- to 60-day-old cotton seedlings grown under ambient and elevated CO_2_ conditions (Table 3).

**FIGURE 4:**
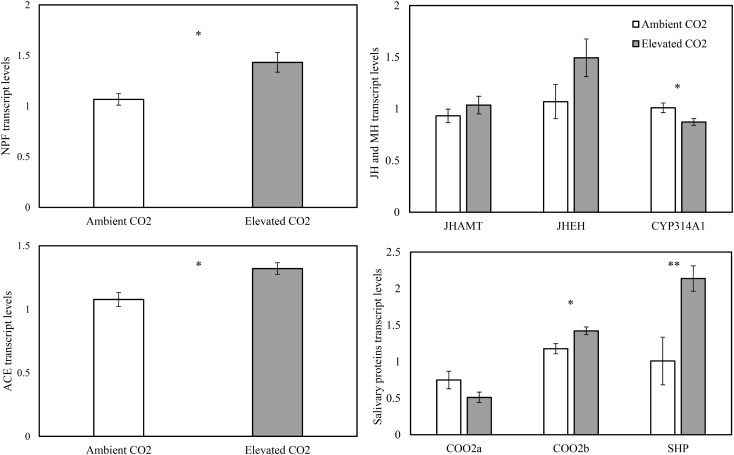
Relative transcript levels (Mean ± SE) of the appetite genes of neuropeptide F (NPF) and angiotensin converting enzyme (ACE), the salivary protein genes of C002a, C002b, and salivary sheath protein (SHP), and the molting hormone (MH) gene of CYP314A1, and the juvenile hormone (JH) (JH) genes of JHAMT and JHEH of the cotton aphid, *Aphis gossypii* fed on the fully expanded leaves excised from the 35- to 60-day-old cotton seedlings grown under ambient and elevated CO_2_ (Asterisks indicate a significant difference between the treatments of ambient and elevated CO_2_ by the LSD test at *P* < 0.05).

As compared with ambient CO_2_, elevated CO_2_ significantly upregulated the relative transcript levels of the salivary protein genes of C002b and SHP by 20.80% and 111.85% and the appetite related genes of NPF and ACE by 34.27% and 22.66%, respectively (P < 0.05), simultaneously, downregulating the relative transcript level of the salivary protein gene of C002a by 31.80% (*P* > 0.05; Figure 4). Moreover, elevated CO_2_ also upregulated the expression levels of the JH genes of JHAMT and JHEH by 11.20% and 39.64%, respectively (*P* > 0.05), simultaneously, significantly downregulating the expression levels of the MH gene of CYP314A1 by 13.60% (*P* < 0.05; Figure 4).

## Discussion

Currently, the global atmospheric CO_2_ concentration continues to rise, standing now at 400 ppm and possibly reaching 800 ppm by the end of this century (Pachauri et al., 2014). As the main factor responsible for global warming, elevated CO_2_ directly induces changes in plant growth, development, metabolism, and plant chemistry (Dader et al., 2016; Jiang et al., 2016); meanwhile, insects are sensitive to these environmental variations, which cause changes in their behavior, growth, development, fertility, and the occurrence of populations as a result of metabolic rate fluctuation (Sun et al., 2015, 2017; He et al., 2017). With the elevated CO_2_ condition, Oehme et al. (2013), in their study, observed that the concentrations of fructose and glucose in spring wheat showed a significant increase, whereas the total amino acid concentration was not altered. These changes in plant chemistry positively affect the relative growth rate (RGR) of aphids. In this study, we found that elevated CO_2_ had significant effects on the soluble nutrients of cotton, which, thereby, were beneficial to the performance of *A. gossypii* because of the bottom-up effects of the plant, which was in accordance with previous studies (Guo et al., 2013, 2014a). Moreover, the qPCR results also indicated that elevated CO_2_ induced a certain degree of upregulation in JH transcription and a significant downregulation in MH transcription, whereas the transcription of genes related to appetite (NPF and ACE) and salivary proteins (C002b and SHP) was significantly upregulated under elevated CO_2_; all these molecular evidences determined here supported our findings well.

In general, elevated atmospheric CO_2_ generally presents positive effects on foliar soluble nutrition of plants, especially in C_3_ plants (Chen et al., 2005a; Wu et al., 2007; Guo et al., 2013). These alterations on the quality of plant host tissue can directly affect the performance of herbivorous insects. However, the response to elevated CO_2_ varies between insects that have piercing and chewing mouthparts (Coll and Hughes, 2008; Sun et al., 2016). A recent meta-analysis examining the effects of elevated CO_2_ on the life history traits of insects found that while the abundance of foliage feeders tends to decrease, phloem feeders on average tend to perform better under elevated CO_2_ (Robinson et al., 2012). Generally, elevated CO_2_ shows negative effects on chewing insects with a decline in the foliar nitrogen content of host plants; as a recent study on the cotton bollworm, *Helicoverpa armigera*, showed that larval durations were significantly prolonged by elevated CO_2_, additionally, female pupal weight, fecundity, and total population size under elevated CO_2_ were lower than ambient CO_2_ (Liu et al., 2017). In contrast, aphids, as a kind of phloem feeders, are considered the only feeding guild that positively responds to elevated CO_2_. Our previous study has shown that, according to four successive generation data, elevated CO_2_ significantly increases fresh body weight, fecundity, and population abundance of *A. gossypii* (Jiang et al., 2016). In regard to *Rhopalosiphum padi* reared on *Hordeum vulgare*, which was maintained under elevated CO_2_, there was a significant increase in aphid abundance and intrinsic rate of population increase; however, there were no statistically significant effects on fecundity and development time of the aphid, such beneficial performance of *R. padi* results from plant biochemical response; under elevated CO_2_ (Ryan et al., 2015). However, piercing-sucking insect seems to have a species-specific response; in terms of studying the population responses of five aphid species to elevated CO_2_, one species showed an increase (*Myzus persicae*), one showed a decrease (*A. pisum*), and the other three remained unaffected (*Aphis nerii*, *A. oenotherae*, *Aulacorthum solani*) (Hughes and Bazzaz, 2001). *Bemisia tabaci* under elevated CO_2_ treatment had a neutral response with no alterations in its life span, sex ratio, and fecundity (Sun et al., 2011). So, as CO_2_ is the substrate for plant photosynthesis, elevated CO_2_ may directly alter physiological and biochemical processes in plants. Furthermore, this indirectly affects insect physiological metabolism by changing plant nutrition and plant defense (Todgham and Stillman, 2013; Guo et al., 2014a,b).

According to our study on the expression of key genes of the JH and MH pathways, it indicated that elevated CO_2_ slightly decreased MH transcription and mildly increased JH transcription. The JH and the main ecdysteroid (20E), known as highly versatile hormones, regulate many aspects of insect physiology, such as development, growth, reproduction, and aging (Riddiford, 1994; Flatt et al., 2008). Recent research suggested that JH was also involved in the regulation of final insect size and growth rates (Mirth et al., 2014). Studies on the tobacco hornworm *Manduca sexta* showed that a decline in circulating JH initiates the first step in the hormonal cascade that begins with the attainment of critical weight, and ends, after a terminal growth period (TGP), with the rise in circulating ecdysone that stops body growth (Fain and Riddiford, 1975; Cymborowski et al., 1982). Intriguingly, similar result was observed in *D. melanogaster*; additionally, it was demonstrated that the effect of JH on growth rate and final body size was mediated by ecdysone synthesis via the regulation of the insulin/insulin-like growth factor (IGF) signaling (IIS) pathway by JH, without affecting the developmental timing (Colombani et al., 2005; Mirth et al., 2014). Our research speculated that JH might be cooperating with MH on regulating MRGR in *A. gossypii* through the key effector of the IIS pathway, like the results obtained of Mirth et al. (2014). These data were in line with previous studies that speculated a cross talk between JH and IIS in *A. gossypii*.

In this study, for the purpose of matching the higher growth rate observed under elevated CO_2_, *the A. gossypii* aphids needed to increase their food intake to obtain enough nutrition for growth. So, our EPG recordings showed that *the A. gossypii* aphids had higher efficiency of stylet penetration under elevated CO_2_ compared with ambient CO_2_. In our previous study, one reason for this result might be that the increase in leaf turgor and soluble constituents of the leaf favored ingestion in *A. gossypii* (Jiang et al., 2016). Sun et al. (2015) also provided the evidence for increased ingestion under elevated CO_2_; that is, originally, *A. pisum* infestation triggered the abscisic acid (ABA) signaling pathway to decrease the stomatal apertures of *Medicago truncatula*, which consequently decreased leaf transpiration and helped to maintain the leaf water potential. Furthermore, elevated CO_2_ upregulates an ABA-independent enzyme, carbonic anhydrase, which led to a further decrease in the stomatal aperture of aphid-infested plants. Thus, the effects of elevated CO_2_ accentuated stomatal closure and synergistically increased leaf turgor in plants, resulting in enhanced aphid feeding. The second case might be that elevated CO_2_ alters plant resistance. For piercing-sucking insects, the results obtained by Sun et al. (2013) indicated that the JA-regulated defense against M. persicae was more effective than the SA-regulated defense in *Arabidopsis* and that elevated CO_2_ tends to enhance the ineffective SA signaling pathway and reduce the effective JA signaling pathway against aphids. Later, similar studies in regard to *A. pisum* reared on *M. truncatula* also demonstrated that elevated CO_2_ enhances the SA-dependent defense pathway and suppresses the JA/ethylene-dependent defense pathway (Guo et al., 2014b, 2016). A recent research on elevated plant resistance in response to CO_2_ indicated that the heat shock protein 90 plays a critical role in plant resistance against the aphid under elevated CO_2_ (Sun et al., 2017). Taken together, elevated CO_2_ increased host water potential and decreased plant resistance against piercing-sucking insects, which favored ingestion and growth of *A. gossypii*.

With respect to the appetite of *A. gossypii*, our results indicated that elevated CO_2_ significantly increased aphid appetite and further regulated the feeding behavior. As known, SHP and C002 play important roles in the stylet probing phase and phloem feeding phase, respectively, in piercing-sucking insects. Insect stylet movement is accompanied by the secretion of gel saliva, which forms a salivary flange on the epidermis and an enveloping salivary sheath in the apoplast, both of which may provide stability, lubrication, and protection during feeding, while the latter also seals the plasma membrane at stylet penetration sites (Will and van Bel, 2006; Will et al., 2012). Abdellatef et al. (2015) showed that silencing the expression of SHP causes transgenerational feeding suppression in *Sitobion avenae*, additionally, reduced SHP expression correlates with a decline in growth, reproduction, and survival rates. The *A. pisum* aphids inject the protein C002 into the host plant during feeding to increase the acquisition of phloem sap. Knockdown of C002 in this aphid causes a decrease in the time that it spends in contact with the phloem sap (Mutti et al., 2008). In the current experiment, our results have revealed for the first time that the increased expression of the salivary protein genes was induced by the high expression of the insect appetite related genes and that this then leads to the higher efficiency of ingestion under elevated CO_2_ condition. But, in the present study, still only little was known about how elevated CO_2_ impacts the appetite of aphids; it needs further studies in the future. Overall, elevated CO_2_ could increase plant phloem nutrition, which, in turn, favored the fitness of aphids via enhanced ingestion due to improved appetite. All the supporting evidences might point to the fact that the rising CO_2_ concentration increases the risk of pest control under the conditions of climate change in the future.

## Author Contributions

All the authors listed have made a substantial, direct, and intellectual contribution to the work and have approved its publication. SJ and FC designed the study. SJ, YD, YqL, and YmL performed the experiments. SJ and FC analyzed the data. SJ wrote the manuscript. SJ, FC, MP, SF, and MB reviewed and polished the manuscript.

## Conflict of Interest Statement

The authors declare that the research was conducted in the absence of any commercial or financial relationships that could be construed as a potential conflict of interest.
